# Alkaloids and Sesquiterpenes from the South China Sea Gorgonian *Echinogorgia pseudossapo*

**DOI:** 10.3390/md9112479

**Published:** 2011-11-24

**Authors:** Cheng-Hai Gao, Yi-Fei Wang, Shen Li, Pei-Yuan Qian, Shu-Hua Qi

**Affiliations:** 1 Key Laboratory of Marine Bio-resources Sustainable Utilization, Guangdong Key Laboratory of Marine Materia Medica, RNAM Center for Marine Microbiology, South China Sea Institute of Oceanology, The Chinese Academy of Sciences, 164 West Xingang Road, Guangzhou 510301, China; E-Mail: gaochenghai@yahoo.com.cn; 2 Guangzhou Jinan Biomedicine Research and Development Center, Guangzhou 510632, China; E-Mails: twangyf@jnu.edu.cn (Y.-F.W.); bghaishen@qq.com (S.L.); 3 Department of Biology, Hong Kong University of Science and Technology, Clearwater Bay, Kowloon, Hong Kong, China; E-Mail: boqianpy@ust.hk

**Keywords:** *Echinogorgia pseudossapo*, gorgonian, zoanthoxanthin alkaloid, sesquiterpene

## Abstract

Five zoanthoxanthin alkaloids (**1**–**5**) and four sesquiterpenes (**6**–**9**) were isolated from the South China Sea gorgonian *Echinogorgia pseudossapo*. Their structures were determined on the bases of extensive spectroscopic analyses, including 1D and 2D NMR data. Among them, pseudozoanthoxanthins III and IV (**1**–**2**), 8-hydroxy-6β-methoxy-14- oxooplop-6,12-olide (**6**) and 3β-methoxyguaian-10(14)-en-2β-ol (**7**) were new, **1** and **3** showed mild anti-HSV-1 activity, and **7** showed significant antilarval activity towards *Balanus amphitrite* larvae.

## 1. Introduction

**Figure 1:**
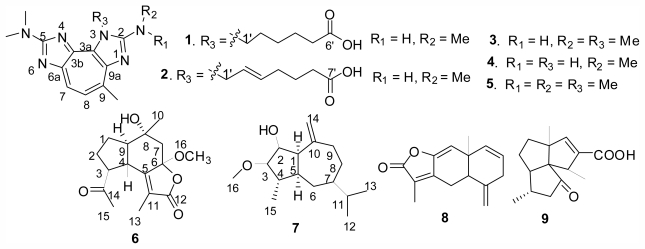
Structures of compounds **1**–**9**.

Gorgonian *Echinogorgia pseudossapo* belongs to the genus *Echinogorgia* that is known to produce sesquiterpenes and sterols [1,2]. The zoanthoxanthins are unusual non-benzenoid aromatic zoochromic alkaloids, which have been isolated exclusively from colonial anthozoans in both major families (Epizoanthidae and Zoanthidae) of the order Zoanthidea, and appeared as three types of skeletons including 3*H*-zoanthoxanthin, 4*H*-pseudozoanthoxanthin, and 3*H*-pseudozoanthoxanthin [3–6]. Some of them showed histamine-like action on the guinea-pigileum and papaverine-like bioactivities [5]. During the course of our series investigations on the chemical constituents of the South China Sea gorgonian corals, five zoanthoxanthin alkaloids (**1**–**5**) and four sesquiterpenes (**6**–**10**) were obtained from the EtOH/CH_2_Cl_2_ extract of the South China Sea gorgonian *E. pseudossapo.* Among these compounds, pseudozoanthoxanthins III–IV (**1**–**2**) [7], 6β-methoxy-14-oxo-oplopa-8α-ol-6,12-olide (**6**) and 3β-methoxy-guaia-2β-ol-10(14)-ene (**7**) were new, and the known compounds were identified as zoanthoxanthin 1 (**3**) [4], paragracine (**4**) [4], zoanthoxanthin (**5**) [4], dehydrolindestrenolide (**8**) [8], and subergorgic acid (**9**) [9] (Figure 1). The antiviral activity of **1**–**4** against herpes simplex virus type 1 (HSV-1) and antilarval activity of **7** towards *Balanus amphitrite* larvae were evaluated. In this paper, we report the isolation, structure elucidation, and bioactivities of these new compounds.

## 2. Results and Discussion

**Figure 2:**
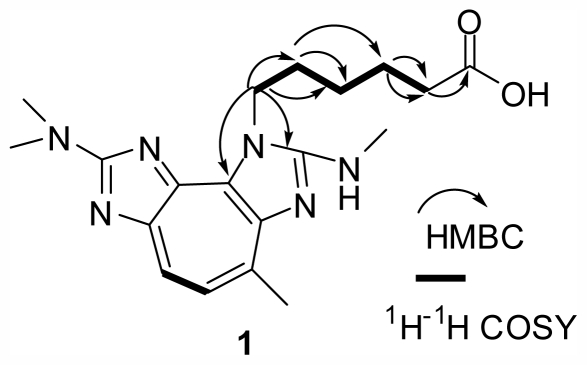
Key ^1^H–^1^H COSY and HMBC correlations of compound **1**.

Compound **1** had a molecular formula of C_19_H_26_N_6_O_2_ deduced from its ESIMS and NMR data. The ^1^H and ^13^C NMR spectra of **1** were similar to those of pseudozoanthoxanthin A [3], pseudozoanthoxanthins I and II [6,10], zoanthoxanthin 1 (**3**) [4], paragracine (**4**) [4] and zoanthoxanthin (**5**) [4] (Table 1), except for the addition of five methylene units and one carboxyl group (δ_C_ 176.4), which suggested that **1** has the same 3*H*-pseudozoanthoxanthin core as **4**, the difference between them existing in the side chain. The HMBC spectrum of **1** (Figure 2) showed correlations of H-1′ (δ_H_ 3.20, t, *J =* 6.5 Hz) with C-2′ (δ_C_ 29.7)/C-3′ (δ_C_ 26.9), H-2′ (δ_H_ 1.54, m) with C-1′ (δ_C_ 39.9)/C-3′/C-4′ (δ_C_ 26.3), H-3′ (δ_H_ 1.35, m) with C-1′ (δ_C_ 39.9)/C-2′/C-4′/C-5′ (δ_C_ 36.7), H-4′ (δ_H_ 1.65, m) with C-3′/C-5′/C-6′ (δ_C_ 176.4), H-5′ (δ_H_ 2.21, t, *J =* 7.5 Hz ) with C-3′/C-4′/C-6′ (δ_C_ 176.4), which suggested the presence of an –N–CH_2_–CH_2_–CH_2_–CH_2_–CH_2_–COOH unit. The suggestion was supported by the ^1^H–^1^H COSY spectrum (Figure 2) showing correlations of H-2′ with H-1′/H-3′, and H-4′ with H-3′/H-5′, and the ESIMS (positive) spectrum showing a main fragment ion peak at *m/z* 257 {100%, [M + 2H–(CH_2_–CH_2_–CH_2_–CH_2_–CH_2_–COOH)]^+^}. The weak HMBC correlations of H-1′ with C-2 (δ_C_ 160.8, s)/C-3a (δ_C_ 132.3, s) and comparison of the ^13^C NMR data of C-3a in **1** and **4** (Table 1) suggested that the –CH_2_–CH_2_–CH_2_–CH_2_–CH_2_–COOH unit should be attached on the nitrogen atom N(3) instead of the another nitrogen atom attached at C(2). So, the structure of **1** was determined as shown and the compound was named pseudozoanthoxanthin III.

Compound **2** had a molecular formula of C_20_H_26_N_6_O_2_ deduced from its (−) ESIMS spectrum (*m/z* 381 [M − H]^−^) and NMR spectra. Comparison of ^1^H and ^13^C NMR spectral data (Table 1) revealed close similarities between **2** and **1**. The difference between them was the absence of one methylene group and the appearance of a 1,2-disubstituted double bond [δ_H_ 5.59 (1H, dd, *J* = 6.5, 16.0 Hz), 5.50 (1H, m), δ_C_ 130.8, 131.8]. Extensive 2D NMR analyses, including HSQC, HMBC and ^1^H–^1^H COSY spectra proved that **1** and **2** had the same skeleton. Moreover, the HMBC spectrum showed correlations of H-1′ (δ_H_ 4.14) with C-2′ (δ_C_ 130.8)/C-3′ (δ_C_ 131.8), H-2′ (δ_H_ 5.59) with C-1′ (δ_C_ 58.6)/C-3′/C-4′ (δ_C_ 27.7), H-3′ (δ_H_ 5.50) with C-1′ (δ_C_ 58.6)/C-2′/C-4′/C-5′ (δ_C_ 25.9), H-4′ (δ_H_ 2.14) with C-3′/C-5′/C-6′ (δ_C_ 34.3), H-5′ (δ_H_ 1.69) with C-2′/C-3′/C-4′/C-6′, and H-6′ (δ_H_ 2.31) with C-4′/C-5′/C-7′ (δ_C_ 177.6), which suggested the presence of an –N–CH_2_–CH=CH– CH_2_–CH_2_–CH_2_–COOH unit.

This suggestion was supported by the ^1^H–^1^H COSY spectrum (Figure 2) showing correlations of H-2′ with H-1′/H-3′, H-4′ with H-3′/H-5′, and H-5′ with H-6′, and the (−) ESIMS spectrum showing one main fragment ion peak at *m/z* 255. In the ^1^H NMR spectrum of **2**, the coupling constant of H-2′/H-3′ (*J* = 16.0 Hz) indicated that geometric configuration of double bond H-2′/H-3′ was *E*. The weak HMBC correlations of H-1′ with C-2 (δ_C_ 160.8, s)/C-3a (δ_C_ 131.9, s) and comparison of the ^13^C NMR data of C(3a) in **2** and **4** (Table 1) suggested that the –CH_2_–CH=CH–CH_2_–CH_2_–CH_2_–COOH unit should be attached to the nitrogen N(3). Thus, the structure of **2** was determined as shown and named pseudozoanthoxanthin IV.

**Figure 3:**
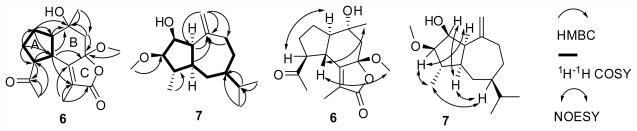
Key HMBC, ^1^H–^1^H COSY and NOESY correlations of compounds **6** and **7**.

Compound **6** had the molecular formula of C_16_H_22_O_5_ as deduced from EIMS and NMR spectra. Its ^1^H NMR spectrum displayed four methyls at δ_H_ 1.80 (3H, s), 1.36 (3H, s), 2.26 (3H, s), 3.14 (3H, s). The ^13^C and DEPT-135 NMR spectra showed 17 carbons consisting of four methyls (δ_C_ 8.7, 21.9, 28.7, 50.0), three methylenes (δ_C_ 23.4, 27.0, 51.2), three methines (δ_C_ 42.1, 52.1, 56.7), two oxygenated quaternary carbons (δ_C_ 71.5, 106.9), one double bond (δ_C_ 121.4, 157.5), one lactone group (δ_C_ 171.9), and one keto group (δ_C_ 208.2). The ^1^H and ^13^C NMR spectral data of **6** showed similarity to those of 7β-hydroxyoplop-11-enone [11] and 7β-senecioyloxyoplopa-3(14)*Z*,8(10)-dien-2-one [12], which suggested that **6** was an oplopane-type sesquiterpene. The suggestion was confirmed by the HMBC and ^1^H–^1^H COSY spectra. In the HMBC spectrum (Figure 3), correlations of H-4 (δ_H_ 2.65, dd, *J* = 11.0, 12.5 Hz) with C-5 (δ_C_ 157.5)/C-6 (δ_C_ 106.9)/C-8 (δ_C_ 71.5)/C-11(δ_C_ 121.4, s), H-7 (δ_H_ 2.53, 1.77, each 1H, d, *J* = 13.5 Hz) with C-6/C-8/C-9 (δ_C_ 56.7), H-9 (δ_H_ 1.84, 1H, m) with C-4 (δ_C_ 42.1)/C-5/C-8, and H-13 (δ_H_ 1.80, 3H, s) with C-5/C-11/C-12 (δ_C_ 171.9, s), suggested the presence of the B,C-ring substructure and Me-13 attached on C-11 to form a methyl substituted α,β-unsaturated γ-lactone unit. In addition, HMBC correlations of H-10 (δ_H_ 1.36, 3H, s) with C-7/C-8/C-9, and H-16 (δ_H_ 3.14, 3H, s) with C-6 (δ_C_ 106.9) indicated that Me-10 and OMe-16 were connected with C-8 and C-16, respectively. Meanwhile, the ^1^H–^1^H COSY spectrum (Figure 3) showed correlations of H-1 [δ_H_ 1.94, 1.63 (each 1H, m)] with H-9/H-2 [δ_H_ 2.28, 1.77 (each 1H, m)], and H-3 (δ_H_ 3.31, ddd, *J* = 8.5, 11.0, 16.8 Hz) with H-2/H-4, suggesting the presence of A-ring unit. The suggestion was supported by HMBC correlations of H-1 with C-4/C-8/C-9, H-2 with C-1 (δ_C_ 23.4)/C-3 (δ_C_ 52.1)/C-4/C-9, and H-3 with C-2 (δ_C_ 27.0)/C-4. Furthermore, HMBC correlations of H-15 (δ_H_ 2.26, 3H, s) with C-3/C-14 (δ_C_ 208.2), and H-3 with C-14 indicated that an acetyl group was attached on C(3).

The relative stereochemistry of **6** was deduced from the NOESY spectrum (Figure 3) and comparison with that of 7β-hydroxyoplop-11-enone [11]. NOE correlations of H-3 with H-9 indicated that H-3 and H-9 were in the same α-oriented direction, and NOE correlations of H-4 with Me-10/Me-16 suggested that H-4, Me-10, and Me-16 were on the same β-oriented side. So, the structure of **6** was elucidated as shown and named 8-hydroxy-6β-methoxy-14-oxooplop-6,12-olide. Oplopanes are frequently found in terricolous plant. However this is the first report of an oplopane-type sesquiterpene isolated from a marine animal.

Compound **7** had the molecular formula of C_16_H_28_O_2_ deduced from NMR spectra and ESIMS. The ^1^H NMR spectrum of **7** displayed signals for four methyls at δ_H_ 0.78 (3H, d, *J* = 6.9 Hz), 0.95 (3H, d, *J* = 6.9 Hz), 1.15 (3H, d, *J* = 6.5 Hz), 3.37 (3H, s) and two oxymethines at δ_H_ 3.67 (1H, dd, *J* = 7.0, 11.0 Hz), 4.15 (1H, dd, *J* = 7.0, 10.8 Hz). The ^13^C NMR spectrum showed 16 carbons including four methyls (δ_C_ 15.8, 22.0, 30.3, 57.1), three methylenes (δ_C_ 24.8, 25.9, 31.5), five high-filed sp^3^ methines (δ_C_ 29.1, 43.7, 44.5, 45.3, 53.3), two oxymethines (δ_C_ 78.3, 90.6), and one double bond [δ_C_ 109.2 (t), 147.8 (s)]. The ^13^C and ^1^H NMR data of **7** were similar to those of guaia-1(10),11-diene and guaia-9,11-diene [13], which suggested that **7** was a guaiane-type sesquiterpene.

The suggestion was supported by ^1^H–^1^H COSY and HMBC spectra (Figure 3). The presence of five membered ring substructure was concluded from the ^1^H–^1^H COSY spectrum showing correlations of H-2 (δ_H_ 4.15, dd, *J* = 7.0, 10.8 Hz) with H-1 (δ_H_ 2.97, t, *J* = 7.0 Hz)/H-3 (δ_H_ 3.67, 1H, dd, *J* = 7.0, 11.0 Hz), H-4 (δ_H_ 1.73, m) with H-3/H-5 (δ_H_ 2.14, m), and H-1 with H-5. The presence of seven membered ring substructure was inferred from the ^1^H–^1^H COSY spectrum showing correlations of H-6 (δ_H_ 1.97, 1.53, each 1H, m) with H-5/H-7 (δ_H_ 1.28, m), H-8 (δ_H_ 1.67, 2H, m) with H-7/H-9 (δ_H_ 2.04, 2.24, each 1H, m), and HMBC spectrum showing correlations C-10 (δ_C_ 147.8) with H-1/H-5/H-9. Furthermore, in the HMBC spectrum, correlations of H-14 [δ_H_ 4.64, 4.62 (each 1H, s)] with C-1 (δ_C_ 53.3)/C-9 (δ_C_ 31.5)/C-10 suggested one double bond between C-10 and C-14. HMBC correlations of H-12 (δ_H_ 0.78, 3H, d, *J* = 6.9 Hz) and H-13 (δ_H_ 0.95, 3H, d, *J* = 6.9 Hz) with C-7 (δ_C_ 43.7)/C-11 (δ_C_ 29.1), and H-11 (δ_H_ 1.73, 1H, m) with C-7/C-12 (δ_C_ 15.8)/C-13 (δ_C_ 22.0) indicated that an isopropyl unit attached on C-7 of the seven membered ring substructure. Meanwhile, HMBC correlations of H-16 (δ_H_ 3.37, 3H, s) with C-3 (δ_C_ 90.6), and H-15 (δ_H_ 1.15, 3H, d, *J* = 6.5 Hz) with C-4 (δ_C_ 44.5), indicated that one methoxy group and one methyl were connected with C-3 and C-4, respectively.

The relative configuration of **7** was determined by a NOESY experiment (Figure 3) and comparison with that of guaia-1(10),11-diene and guaia-9,11-diene [13]. Considering the bulky isopropyl group to keep a *pseudo* equatorial position and being β-oriented, H-7 had to be α-oriented. NOE correlations of H-1 with H-2/H-3/H-5, H-2 with H-5, H-3 with H-5/H-7/Me-15, and H-7 with H-5/Me-15 suggested that H-1, H-2, H-3, H-5, and Me-15 were oriented in the same direction as H-7, and should be α-orientation. Based on the above data, the structure of **7** was determined as shown and named 3β-methoxyguaian-10(14)-en-2β-ol.

*In vitro* antiviral activity of **1**–**4** against HSV-1 was evaluated using plaque reduction assay. First, the completely non-toxic concentration (CC_0_) of **1**–**4** and positive control ACV on Vero cells were tested to be 270.3, 523.6, 185.2, 195.3, >7500 μM by MTT assays, respectively, then for further antivirus studies, the concentrations of tested compounds were kept below their CC_0_ values. The antiviral assays displayed that **1**–**4** exhibited anti-HSV-1 activity with EC_50_ (50% effective concentration required to inhibit virus-induced cytopathicity 50%) values of 108.1, 471.2, 70.4, 117.2, 6.08 μM, respectively. The results suggested that the side chain at the nitrogen N(3) in **1**–**4** could affect their antiviral activity. Although **1** and **3** showed mild anti-HSV-1 activity, their activities were far lower than that of the positive control ACV.

Compound **7** was evaluated for its antilarval activity against *B. amphitrite* and *B. neritina* larvae. The results showed that **7** had significant antilarval activity towards *B. amphitrite* larvae with EC_50_ value of 17.2 μg/mL (68.2 μM), and showed 50% inhibition towards the settlement of *B. neritina* larvae at concentration of 25 μg/mL. The EC_50_ value of **7** is lower than the standard requirement of an EC_50_ of 25 μg/mL established by the US Navy program as an efficacy level for natural antifoulants, indicating that **7** is a potential natural antifouling agent.

## 3. Experimental Section

### 3.1. General

Optical rotations were measured with a Horiba SEAP-300 spectropolarimeter. UV spectra were measured with a Shimadzu double-beam 210A spectrophotometer in MeOH solution. ^1^H, ^13^C NMR and 2D NMR spectra were recorded on a Bruker AV-500 MHz NMR spectrometer with TMS as internal standard. MS spectral data were obtained on an LCQDECA XP HPLC/MSn spectrometer for ESIMS.

### 3.2. Animal Material

The South China Sea gorgonian coral *E. pseudossapo* (7.8 kg, wet weight) was collected in Sanya, Hainan Province, China in October 2007 and identified by Research Assistant Xiubao Li, the South China Sea Institute of Oceanology, Academia Sinica (SCSIO). A voucher specimen (No. 2007-SCSIO-3) was deposited in SCSIO, Guangzhou, China.

### 3.3. Extraction and Isolation

The frozen specimens of *E. pseudossapo* were exhaustively extracted with EtOH/CH_2_Cl_2_ (2:1) three times at room temperature, and the solvent was evaporated in *vacuo*. The residue was partitioned in H_2_O and extracted with EtOAc and *n*-BuOH in turn three times, respectively. The *n*-BuOH extract was concentrated in *vacuo* to afford 10.2 g of residue, and then the *n*-BuOH portion was subjected to column chromatography on silica, using CHCl_3_/MeOH (from 10:0 to 0:10) as eluent. By combining the fractions with TLC (GF254) monitoring, 8 fractions were obtained. Fraction 2 was chromatographed over Sephadex LH-20 eluting with CHCl_3_/MeOH (1:1) to obtain three sub-fractions (A–C). Sub-fraction B was purified over semi-preparative HPLC with MeOH/water (50:50) to yield **1** (10 mg) and **3** (4.0 mg). Sub-fraction C were purified over semi-preparative HPLC eluted with MeOH/H_2_O (60:40) to yield **2** (10.1 mg), **4** (13.0 mg), and **5** (2.3 mg). The EtOAc extracts were concentrated *in vacuo* to afford 33.5 g of residue. The EtOAc portion was subjected to column chromatography (CC) on silica, using petroleum ether-EtOAc (from 10:1 to 0:10) as eluent. By combining the fractions with TLC (GF254) monitoring, 16 fractions were obtained. Fraction 7 was purified by silica gel column, eluted with petroleum ether-EtOAc (2:1) to yield **8** (17.0 mg). Fraction 8 was subjected to CC on silica gel, eluted with CHCl_3_-Me_2_CO (from 100:5 to 0:10), and then purified with semi-preparative HPLC, using MeOH-water as eluent to afford **6** (10.0 mg) and **9** (6.4 mg). Fraction 10 was chromatographed over Sephadex LH-20 eluting with CHCl_3_/MeOH(1:1), then repeatedly subjected to CC on Si gel, eluted with CHCl_3_/MeOH (from 10:0 to 6:4) to yield **7** (10.3 mg).

*Pseudozoanthoxanthin III* (**1**): Yellow oil; UV (MeOH) λ_max_ 221, 257, 304, 362 nm; IR (KBr) ν_max_ 3400, 3300, 1750, 1690, 1620 cm^−1^; ^1^H NMR and ^13^C NMR spectral data, see Table 1; ESI-MS (+) *m/z* 371 [M + H]^+^; HRESIMS *m/z* 371.2159 [M + H]^+^ (calcd for C_19_H_27_N_6_O_2_ 371.2195).

*Pseudozoanthoxanthin IV* (**2**): Yellow oil; UV (MeOH) λ_max_ 221, 257, 304, 362 nm; IR (KBr) ν_max_ 3407, 3313, 1752, 1694, 1623 cm^−1^; ^1^H NMR and ^13^C NMR spectral data see Table 1; ESI-MS(−) *m/z* 381 [M − H] ^−^; HRESIMS *m/z* 381.2075 [M − H] ^−^ (calcd for C_20_H_25_N_6_O_2_, 381.2039).

*8a-hydroxy-6β-methoxy-14-oxooplop-6,12-olide* (**6**): Colorless oil; [α]^25^ _D_ +0.3 (c 0.10, MeOH); UV (MeOH): 225 nm; IR (KBr) ν_max_ 3276, 1723, 1625 cm^−1^; ^1^H NMR(500 MHz, CDCl_3_) δ_H_: 1.94, 1.63 (each 1H, m, H-1), 2.28, 1.77 (each 1H, m, H-2), 3.31 (1H, ddd, *J =* 8.5, 11.0, 16.8 Hz, H-3), 2.65 (1H, *J =* 11.0, 12.5 Hz, H-4), 2.53, 1.77 (each 1H, d, *J =* 13.5 Hz, H-7), 1.84 (1H, m, H-9), 1.36 (3H, s, Me-10), 1.80 (3H, s, Me-13), 2.26 (3H, s, Me-15), 3.14 (3H, s, OMe-16); ^13^C NMR (125 MHz, CDCl_3_) δ_C_: 23.4 (C-1), 27.0 (C-2), 52.1 (C-3), 42.1 (C-4), 157.5 (C-5), 106.9 (C-6), 51.2 (C-7), 71.5 (C-8), 56.7 (C-9), 21.9 (C-10), 121.4 (C-11), 171.9 (C-12), 8.7 (C-13), 208.2 (C-14), 28.7 (C-15), 50.0 (C-16); HR-EI-MS *m/z* 294.1472 [M]^+^ (calcd for C_16_H_22_O_5_, 294.1467).

*3β-methoxyguaian-10(14)-en-2β-ol* (**7**): Colorless oil; [α]^25^ _D_ +0.8 (c 0.10, MeOH); IR (KBr) ν_max_ 3446, 1648, 1456 cm^−1^; ^1^H NMR(500 MHz, CDCl_3_) δ_H_: 2.97 (1H, t, *J =* 7.0 Hz, H-1), 4.15 (1H, dd, *J =* 7.0, 10.8 Hz, H-2), 3.67 (1H, dd, *J =* 7.0, 11.0 Hz, H-3), 1.73 (1H, m, H-4), 2.14 (1H, m, H-5), 1.97, 1.53 (each 1H, m, H-6), 1.28 (1H, m, H-7), 1.67 (2H, m, H-8), 2.04, 2.24 (2H, m, H-9), 1.73 (1H, m, H-11), 0.78 (3H, d, *J =* 6.9 Hz, Me-12), 0.95 (3H, d, *J =* 6.9 Hz, Me-13), 4.64, 4.62 (each 1H, s, H-14), 1.15 (3H, d, *J =* 6.5 Hz, Me-15), 3.37 (3H, s, OMe-16); ^13^C NMR (125 MHz, CDCl_3_) δ_C_: 53.3 (C-1), 78.3 (C-2), 90.6 (C-3), 44.5 (C-4), 45.3 (C-5), 25.9 (C-6), 43.7 (C-7), 24.8 (C-8), 31.5 (C-9), 147.8 (C-10), 29.1 (C-11), 15.8 (C-12), 22.0 (C-13), 109.2 (C-14), 30.3 (C-15), 57.1 (C-16); HR-EI-MS *m/z* 252.2082 [M]^+^ (calcd for C_16_H_28_O_2_, 252.2089).

### 3.4. Viruses and Cells

HSV-1 (15577) strain and Vero cells were obtained from American Type Culture Collection. Cytotoxicity assay and cytopathic effect reduction assay were undertaken with the reported methods [14]. ACV was used as the positive control.

### 3.5. Larval Settlement Bioassays

Antilarval activity of the compounds was evaluated in settlement inhibition assays with laboratory-reared *Balanus amphitrite* and *Bugula neritina* larvae. The procedures were the same as previously reported [15].

## 4. Conclusion

In conclusion, our investigation on the chemical constituents of gorgonian *E. pseudossapo* led to the obtainment of five zoanthoxanthin alkaloids (**1**–**5**) and four sesquiterpenes (**6**–**9**). Among these compounds, **1**, **2**, **6** and **7** were new, **1** and **3** showed moderate anti-HSV-1 and anti-RSV activity, and **7** showed significant antilarval activity towards *B. amphitrite* larvae. The results elucidate the basis of medicinal substances of *E. pseudossapo*, and suggest that **7** is a potential natural antifouling agent.

## Figures and Tables

**Table 1:** ^1^H NMR (500 MHz) and ^13^C NMR (125 MHz) data of 1, 2, 4 (in CD_3_OD, δ in ppm).

Position	1		2		4

	δ_H_ (mult., *J* in Hz)	δ_C_	δ_H_ (mult., *J* in Hz)	δ_C_	δ_C_
2		160.8, C		160.8, C	161.0
3a		132.3, C		131.9, C	140.4
3b		152.5, C		152.7, C	153.1
5		161.3, C		161.5, C	162.1
6a		142.3, C		143.2, C	143.5
7	7.84 (d, 10.3)	121.9, CH	7.87 (d, 9.5)	119.6, CH	119.5
8	7.79 (d, 10.3)	133.1, CH	7.80 (d, 9.5)	133.5, CH	133.1
9		147.7, C		148.4, C	148.0
9a		135.0, C		135.1, C	135.5
2-NMe	3.23 (s)	29.8, CH_3_	3.38 (s)	29.7, CH_3_	29.8
5-NMe	3.38 (s)	38.7, CH_3_	3.33 (s)	38.6, CH_3_	37.8
Me-9	2.86 (s)	23.3, CH_3_	2.85 (s)	23.4, CH_3_	23.4
1′	3.20 (t, 6.5)	39.9, CH_2_	4.14 (d, 6.5)	58.6, CH_2_	
2′	1.54 (tt, 6.5, 7.0)	29.7, CH_2_	5.59 (dd, 6.5, 16.0)	130.8, CH	
3′	1.35 (tt, 7.0, 7.4)	26.9, CH_2_	5.50 (dd, 7.4, 16.0)	131.8, CH	
4′	1.65 (qt, 7.4, 7.5)	26.3, CH_2_	2.14 (dt, 7.4, 7.5)	27.7, CH_2_	
5′	2.21 (t, 7.5)	36.7, CH_2_	1.69 (qt, 7.5, 7.5)	25.9, CH_2_	
6′		176.4, C	2.31 (t, 7.5)	34.3, CH_2_	
7′				177.6, C	

## References

[1] Manzo E., Ciavatta M.L., Gresa M.P.L., Gavagnin M., Villani G., Naikc C.G., Ciminoa G. (2007). New bioactive hydrogenated linderazulene-derivatives from the gorgonian *Echinogorgia complexa*. Tetrahedron Lett.

[2] Tanaka J., Miki H., Higa T. (1992). Echinofuran, a New Furanosesquiterpene from the gorgonian *Echinogorgia praelonga*. J. Nat. Prod.

[3] Braun M., Buchi G., Bushey D.F. (1978). Synthesis of parazoanthoxanthins and pseudozoanthoxanthins. J. Am. Chem. Soc.

[4] Jiménez C., Crews P. (1993). ^13^C-nmr assignments and cytotoxicity assessment of zoanthoxanthin alkaloids from Zoanthid corals. J. Nat. Prod.

[5] Komoda Y., Shimizu M., Ishikawa M. (1984). Structures of biologically active minor bases related to paragracine from *Parazoanthus gracilis* LWOWSKY. Chem. Pharm. Bull.

[6] Schwartz R.E., Yunker M.B., Scheuer P.J. (1979). Pseudozoanthoxanthins from gold coral. Can. J. Chem.

[7] Qi S.H. (2011). Preparison and application of imidazolidinyl alkaloids from gorgonian*Echinogorgia pseudossapo*. Chinese invention patent.

[8] Zhao H.L., Qin T.Z., Shang Y.J., Wang Z.T. (2001). Assignments of ^1^H NMR fingerprint of the root bark of *Celastrus angulatus*. Acta Pharm. Sin.

[9] Amiram G., William F., He C., Clardy J., Wu Z., Yiao Z., Long K. (1985). Subergorgic acid, a novel tricyclopentanoid cardiotoxin from the pacific gorgonian coral *Subergorgia suberosa*. Tetrahedron Lett.

[10] Skropeta D. (2008). Deep-sea natural products. Nat. Prod. Rep.

[11] Kijjoa A., Vieira L.M., Pereira J.A., Silva A.M.S., Herz W. (1999). Further constituents of *Achillea ageratum.*. Phytochemistry.

[12] Yaoita Y., Kamazawa H., Kikuchi M. (1999). Structures of new oplopane-type sesquiterpenoids from the flower buds of *Tussilago farfara* L. Chem. Pharm. Bull.

[13] Hailemichael T., Wilfried A.K., Karl-Heinz K., Bartnik M., Glena K. (2005). Secondary metabolites of *Peucedanum tauricum* fruits. Phytochemistry.

[14] Ma S.C., Du J., But P.P., Deng X.L., Zhang Y.W., Ooi V.E., Xu H.X., Lee S.H., Lee S.F. (2002). Antiviral Chinese medicinal herbs against respiratory syncytial virus. J. Ethnopharm.

[15] Qi S.H., Zhang S., Qian P.Y., Wang B.G. (2008). Antifeedant, antibacterial and antilarval active secondary metabolites from seagrass *Enhalus acoroides*. Bot. Mar.

